# Cytotoxic effect of pyocyanin on human pancreatic cancer cell line (Panc-1)

**DOI:** 10.22038/IJBMS.2018.27865.6799

**Published:** 2018-08

**Authors:** Aylin Moayedi, Jamileh Nowroozi, Abbas Akhavan Sepahy

**Affiliations:** 1 Department of Microbiology, Tehran North Branch, Islamic Azad University, Tehran, Iran

**Keywords:** Apoptosis, Cytotoxic, Panc-1, Pancreatic cancer, Pseudomonas aeruginosa, Pyocyanin

## Abstract

**Objective(s):**

Pyocyanin, a blue-green pigment produced by *Pseudomonas aeruginosa*, interferes with host redox cycles, which can lead to generation of reactive oxygen species and progressive cellular oxidative damage. The aim of this study was to assess the influence of pyocyanin on human pancreatic cancer cell line.

**Materials and Methods:**

Polymerase Chain Reaction (PCR) was applied to confirm the existence of a specific pyocyanin producing gene (phzM). The pigment was then characterized by UV-visible, FT-IR, and HPLC analysis. Panc-1 cells were treated by different concentrations of pyocyanin and their cytotoxic effect as well as the induction of apoptosis/necrosis were evaluated by XTT assay and flow cytometry.

**Results:**

An overnight pyocyanin treatment resulted in significant cell reduction in a concentration-dependent manner. Inhibition rate of 6 mg.ml-1 pyocyanin (the highest concentration) extracted from clinical and soil isolates of *P. aeruginosa* were 98.69±0.23 and 89.88±1.86%, respectively, which decreased as the pyocyanin concentration lessened. Pyocyanin could also induce dose-dependent apoptosis/necrosis in Panc-1 cells after 24 hr.

**Conclusion:**

We reported, for the first time, cytotoxic effects of pyocyanin against human pancreatic cancer cell line. Considering this effect of the pigment, study on pyocyanin as a potential anti-tumor biodrug requires further studies.

## Introduction

*Pseudomonas aeruginosa*, a common opportunistic and nosocomial pathogen, produces a wide variety of pigments as secondary metabolites amongst gram-negative bacteria. These pigments are not essential for bacterial growth and proliferation but for bacterial pathogenicity and biological control (1, 2). Some of which are redox-active phenazine compounds, including pyocyanin, phenazine-1-carboxylic acid (PCA), 1-hydroxy phenazine, and phenazine-1-carboxamide (3). Pyocyanin, the main phenazine pigment in *P. aeruginosa*, is a small diffusible molecule interacting with molecular oxygen intracellularly and disturbs redox cycling. These changes can result in the generation of reactive oxygen species (ROS), such as hydrogen peroxide (H_2_O_2_) and superoxide, and induce considerable oxidative stress and cell damage (4).

The natural ability of pyocyanin in modulating redox cycles and an increase in oxidative stress appears to play an important role in its detrimental effects on host cells. Some of the potentially toxic effects of pyocyanin are: perturbation of cellular respiration, disrupting Ca^2+^ homeostasis, inhibition of epidermal cell growth, secretion of prostacyclin from lung endothelial cells, and altered balance of protease/anti-protease activity in cystic fibrosis lung (1, 5). In neutrophils, the presence of pyocyanin leads to a persistent increase in ROS and subsequently a considerable decrease in intracellular cAMP, which can be expected to follow by a time- and concentration-dependent acceleration of apoptosis (6).

A number of phenazines, however, do not exert cytotoxic effects in eukaryote cells and have been considered as anti-cancer or anti-infective agents (7, 8). Phenazine compounds show a more powerful interference in actively respiring cells, such as tumor cells. It has also been found that phenazine derivatives can interfere with the function of topoisomerase I and II in eukaryotic cells and since cancer cells contain high levels of topoisomerases, the activity of these enzymes and consequently the proliferation of tumor cells can be affected by pyocyanin. Zhao *et al.* and Hassani *et al.* have recently described the anti-cancer effect of pyocyanin on human hepatoma (HepG2) and rhabdomyosarcoma (RD) cell lines (9, 10). 

Pancreas cancer is among cancers with the highest mortality rates; only 6% of pancreatic cancer patients survive more than five years and most of them die within the first year after diagnosis (11). Progress in diagnosis and treatment of pancreatic cancer is generally disappointing. Current treatment options include surgery, radiotherapy, and chemotherapy, which occasionally may extend survival or decrease symptoms, but can never be regarded as a cure. Lacking progress in prevention, detection, and treatment of pancreatic cancer highlights the need for further research on this subject (12).

Development of phenazine derivatives with anti-cancer activity is a potential research field aiming to link the biological activity of phenazines to particular cancer cells as targets (13). Few are the studies reporting anti-cancer effects of pyocyanin on several cancer cell lines; therefore, the aim of this study was to identify the cytotoxic effect of the pigment on human pancreatic cancer cell line (Panc-1) as well as its role in the induction of apoptosis.

## Materials and Methods

***Clinical and soil samples***

A total of 11 *P. aeruginosa* isolates (10 isolates from wound specimens and one isolate from urinary tract infection, C_1_-C_11_) were kindly donated by the lab of Shaheed Motahari Burns Hospital, Tehran, Iran. Soil samples, additionally, contained 10 *P. aeruginosa* strains (E_1_-E_10_) isolated from ten agricultural and oil-contaminated samples (taken from regions with the lowest risk of hospital specimen contamination) by using the methods described in our previous study (14). All isolates were identified as *P. aeruginosa* by biochemical tests. To select pyocyanin producing strains, isolates were cultured on cetrimide agar and incubated for 48 hr at 37 °C and those produced the darkest green color on the medium were chosen for further assessments.

***Molecular characterization***

Polymerase Chain Reaction (PCR) of a specific pyocyanin biosynthesis gene, *phzM*, was done to confirm pyocyanin production in C_11_ and E_8_ isolates (darkest greenish pigment producers) along with a standard strain (*P. aeruginosa *ATCC 9027). Moreover, an isolate (C_1_), which could produce yellowish but not blue-green pigment was chosen to verify that the *phzM* gene plays a key role in pyocyanin production.

Genomic DNA of overnight broth cultures was extracted by a DNA extraction kit (MBST, Iran) according to the manufacturer’s protocol. PCR mixture contained 18 μl nuclease free water, 2.5 μl of 10× PCR buffer, 1 μl dNTP Mix (Metabion, Germany), 1 μl (4 μM) of each primer, 1 μl of template DNA, and 0.5 μl Taq DNA polymerase (Metabion, Germany). PCR was performed by a gradient thermal cycler (Corbett Palm-Cycler) using the following conditions: one cycle of initial denaturation at 94 °C for 5 min followed by 30 cycles of denaturation at 94 °C for 1 min, annealing at 55.9 °C for 45 sec, extension at 72 °C for 1 min, and the process was finished by the final extension (72 °C for 5 min). Finally, 4 μl of the PCR product was examined by gel agarose electrophoresis against 1 kb DNA marker (Metabion, mi-8201, Germany).

The *phzM* primers generating a 1015 bp DNA fragment used in the present study were:

Forward: 5ʹ-TTTTTCATATGAATAATTCGAATCTTGCTG-3ʹ

Reverse: 5ʹ-TTTTTGGATCCGTTGAAAGTTCCGATTCA-3ʹ (15)

***Pyocyanin production and extraction***

Equal volumes of bacterial suspension from overnight colonies of C_11_ and E_8_ isolates on glycerol-alanine agar (per liter: 10 ml glycerol, 6 g L-alanine, 2 g MgSO_4_, 0.1 g K_2_HPO_4_, 0.018 g FeSO_4_, 20 g agar, final pH 7.2) were inoculated in glycerol-alanine broth separately and incubated for 72 hr at 37 °C with 230-rpm shaking. Thereafter, a 15-min centrifuge at 4000 rpm was done to remove the bacterial cell pellet and the supernatant was filter sterilized for the extraction of pyocyanin by the chloroform-acid extraction procedure as follows:

Chloroform (Merck) was added to the collected supernatant in 1:2 ratio. After a short vortex and centrifuging at 3000 rpm for 1 min, the top layer was removed and the volume of the blue bottom layer (chloroform + pyocyanin) was measured. The extraction followed by adding 20% (v/v) 0.1 N HCl to the blue mixture and after a momentous vortex and centrifuging at 3000 rpm for 1 min, the acidified top pink-red phase was collected in a new tube and neutralized by 0.5 mol.l^-1^ NaOH. Further extraction was performed by the repetition of above steps three times to gain a pigment with high purity. After the adjustment of final pH to 7.5, the solution was filtered and dried in order to form pyocyanin blue needle-like crystals.

***Characterization of pyocyanin***

*UV-visible spectrophotometry*

Pyocyanin was dissolved in chloroform or 0.1 N HCl and UV-Vis absorption spectrum of the pigment was recorded over the range of 300–800 nm by a UV-Vis spectrophotometer (Cary 100 Bio).

*FT-IR spectroscopy*

Fourier Transform Infrared spectroscopy of pyocyanin crystals was performed in 4000 ~ 400 cm^-1^ region of a FT-IR spectrometer (Thermo Scientific Nicolet 8700).

*HPLC*

C_11_ and E_8_ pyocyanin samples were determined against a standard pyocyanin (Sigma-Aldrich P0046, USA) by High-Performance Liquid Chromatography analyses. The isocratic system of HPLC was performed by a Knauer liquid chromatograph (Germany) consisting of a WellChrom HPLC pump K-1001 with a Reodyne 20 μl injector model 7125, a UV detector K-2501 set at 375 nm, and a Shimadzu C-R5A Chromatopac data processor. A 5-μm particle size C18 column (250 × 4.6 mm) was used and the mobile phase was 100% acetonitrile. The flow rate was 1 ml.min^-1^ and the column temperature was maintained at 30 ^°^C.

***Cell culture***

Panc-1 cell line was provided by Pasteur Institute of Iran and cultured in DMEM-high glucose medium supplemented with 10% fetal bovine serum (Biosera, UK) and 1% penicillin-streptomycin solution. Prepared 24- and 96-well plates, containing (per well) 500 μl of 1 × 10^5^ and 100 μl of 1 × 10^4^ Panc-1 cells in DMEM medium, respectively, were incubated for 24 hr at 37 °C in an incubator supplemented with 5% CO_2_. After the incubation period, the medium was replaced with a fresh one.

***XTT cell proliferation assay***

Cells of the 96-well plate were treated by different concentrations of pyocyanin extracted from C_11_ or E_8_ strains (6, 3 mg.ml^-1^, 300, 30, 3 μg.ml^-1^, and 300 ng.ml^-1^ dissolved in DMSO in a volume of 100 μl DMEM medium) in triplicate, and untreated, 1 μl DMSO-exposed cells, were used as control. 

The plate was then incubated for 24 hr at 37 °C with 5% CO_2_. After the incubation time, XTT assay was performed by an XTT cell proliferation kit (Roche, USA) and 50 μl of the XTT solution was added to each well. The plate was incubated for a further 4 hr and thereafter, the absorbance of each well was measured by using a microplate reader at 450 nm wavelength. Inhibitory rate (%IR) of each treatment was calculated as follows %IR = A-B/A×100, where A represents the absorbance of the control, while B represents the absorbance of the treatment (10). IC_50_ of each pigment was measured using the logarithmic scale of %IR on the Y-axis and the concentration of the pigment on the X-axis using the GraphPad Prism 6 software.

***Flow cytometry***

Cells of the 24-well plate were treated by different concentrations of C_11_ or E_8_ pyocyanin (3, 1.5 mg.ml^-1^, 300, 150, and 30 μg.ml^-1^ dissolved in DMSO in a volume of 500 μl DMEM medium), and untreated, 1 μl DMSO-exposed cells, were used as control. Plates were incubated for 24 hr at 37 °C with 5% CO_2_. Annexin/PI staining was done by Annexin-V-Flus Staining Kit (Roche, USA) according to the manufacturer’s protocol and apoptosis and/or necrosis induced by each pigment was examined by a flowcytometer (Partec PAS).

***Statistical analysis***

All data given as mean ± SD was statistically analyzed using Statistical Package for Social Sciences (SPSS version 22.00). Treatment means were compared at 5% level of significance.

## Results

***Selection of the highest pyocyanin producers***

Isolates C_11_ (from urinary tract infection) and E_8_ (obtained from oil-contaminated soil) were the highest pyocyanin producers amongst clinical and soil isolates, respectively. Other isolates produced pigments in yellow, red, or light green.

**Figure 1:**
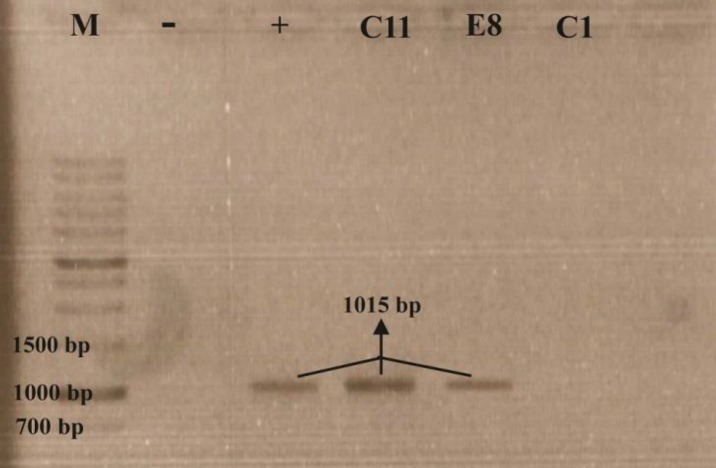
*phzM* bands on gel agarose, (M): DNA marker, (-): negative control, (+): positive control/ P. aeruginosa ATCC 9027

**Figure 2:**
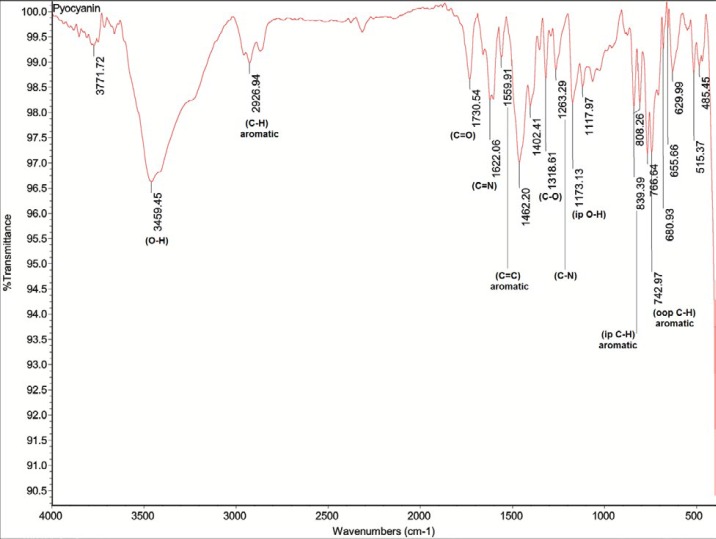
FT-IR spectrum of C_11_ pyocyanin (C_13_H_10_N_2O_)

***Molecular identification of phzM***

C_11_ and E_8_ isolates along with the standard strain contained the 1015-bp DNA fragment of *phzM*, but C_1 _isolate (yellowish pigment producer) could not show the *phzM* band (Figure 1).

***Pyocyanin production and extraction***

C_11_ and E_8_ isolates, which produced dark green pyocyanin pigment on cetrimide agar could produce dark blue pyocyanin pigment on glycerol-alanine agar. Furthermore, of each 150 ml glycerol-alanine broth inoculated by C_11_ or E_8 _isolates, 90 and 75 mg needle-like pyocyanin crystals were extracted, respectively.

***Characterization of pyocyanin***

UV-Vis spectra of pyocyanin exhibited absorption maxima at 308 and 694 nm when dissolved in chloroform, where as values were 387 and 518 nm when dissolved in 0.1 N HCl. 

**Figure 3:**
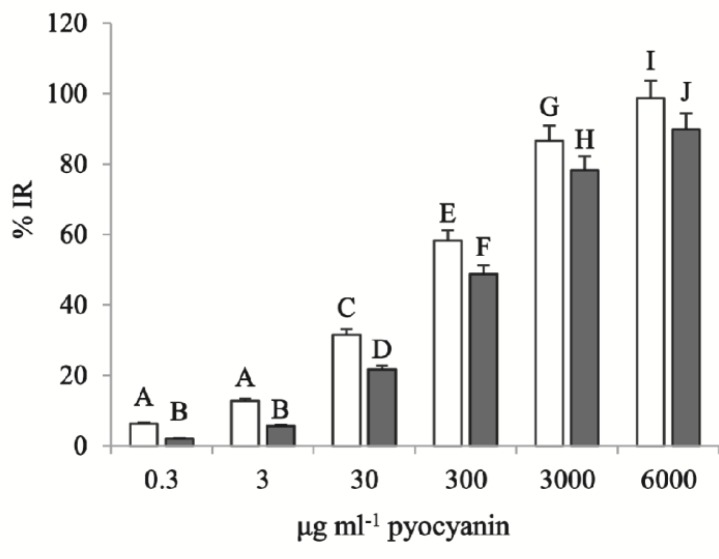
T-test analysis of the comparison between C_11_ and E_8_ pyocyanin cytotoxicity. C_11_ pyocyanin showed higher cytotoxicity than E_8_ with significant differences in all treated groups. Several letters represent in- and between-group significant differences (*P*<0.05)

**Figure 4:**
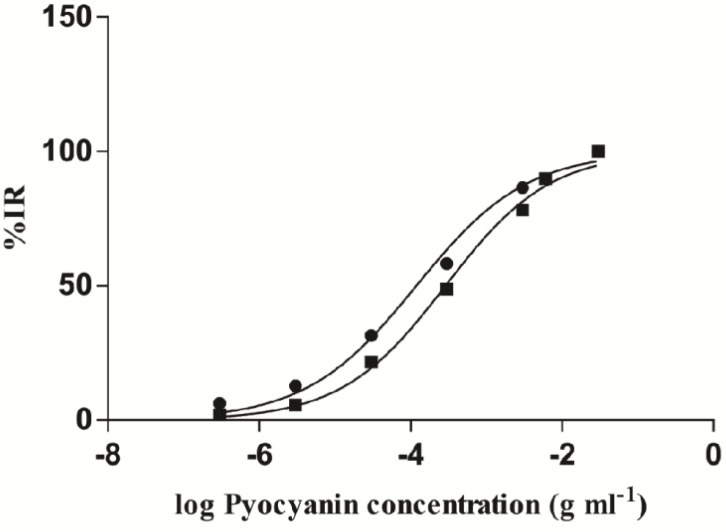
Logarithmic scales of C_11 _and E8 pyocyanin concentrations against %IR. IC50 of C_11_ pyocyanin calculated by the GraphPad Prism software showed a greater result than E_8_ with significant difference (*P* < 0.0001)

**Figure 5:**
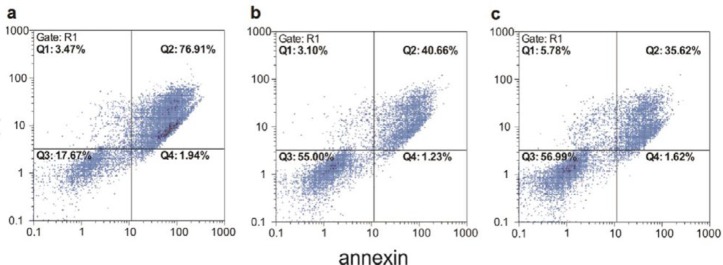
Dot plot results of annexin/PI staining of Panc^-1^ cells treated by (a) 1.5 mg.ml^-1^ (b) 300 μg.ml^-1^ (c) 150 μg.ml-1 of C_11_ pyocyanin that induced apoptosis and/or necrosis in 82.33, 45, and 43.01% of Panc^-1^ cells, respectively. Q_1_: necrotic cells, Q_2_: apoptotic and necrotic cells, Q_3_: viable cells, Q_4_: apoptotic cells

**Table 1:** The proportion of apoptotic and/or necrotic cells. "annexin+" represents apoptotic cells, while "PI+" represents necrotic cells

		annexin-/PI+	annexin+/PI+	annexin+/PI-	annexin-/PI-
C_11_ pyocyanin	3 mg.ml^-1^	1.54%	93.57%	2.69%	2.20%
	1.5 mg.ml^-1^	3.47%	76.91%	1.94%	17.67%
	300 μg.ml^-1^	3.10%	40.66%	1.23%	55.00%
	150 μg.ml^-1^	5.78%	35.62%	1.62%	56.99%
	30 μg.ml^-1^	6.24%	24.49%	1.78%	67.49%
E_8_ pyocyanin	3 mg.ml^-1^	14.09%	55.30%	3.64%	26.96%
	1.5 mg.ml^-1^	8.47%	61.77%	1.24%	28.52%
	300 μg.ml^-1^	5.50%	33.51%	9.71%	51.21%
	150 μg.ml^-1^	11.54%	14.97%	1.48%	72.00%
	30 μg.ml^-1^	3.59%	8.86%	2.59%	84.96%
Untreated Cells		2.43%	0.12%	0.01%	97.45%

The FT-IR spectrum of pyocyanin, as shown in Figure 2, was also in accord with the pyocyanin structure (C_13_H_10_N_2_O).Furthermore, the peak with retention time of 4.766 min represented the standard pyocyanin using HPLC and typical peak maxima were also exhibited by both C_11_ and E_8_ pyocyanin.

***Pyocyanin cytotoxicity***

XTT assay revealed that the highest concentration (6 mg.ml^-1^) of C_11_ and E_8_ pyocyanin led to 98.69±0.23 and 89.88±1.86% reduction of Panc-1 cells, respectively. As the pyocyanin concentration decreased, so did the percentage of cell reduction, and %IR of the lowest concentrations of C_11_ and E_8_ pyocyanin (300 ng.ml^-1^) were 6.25±1.55 and 2.08±1.03%, respectively. All treatments by C_11_ pyocyanin, additionally, resulted in a greater %IR than E_8_ pyocyanin (Figure 3). Inhibitory Rate for the remaining concentrations (3 mg.ml^-1^, 300, 30, and 3 μg.ml^-1^) were 86.61±3.22, 58.33±3.61, 31.54±3.61, and 12.80±1.36 for C_11_ treated groups and 78.28±1.86, 48.81±3.14, 21.73±1.36, and 5.65±1.03 for E_8_ treated groups, respectively. As Figure 4 also shows, IC_50_ of C_11_ pyocyanin calculated by the GraphPad Prism software as 118.5±0.06 μg.ml^-1^, significantly differs from that of E_8_ pyocyanin, which was 287.4±0.04 μg.ml^-1^ (*P*<0.0001).

**Figure 6:**
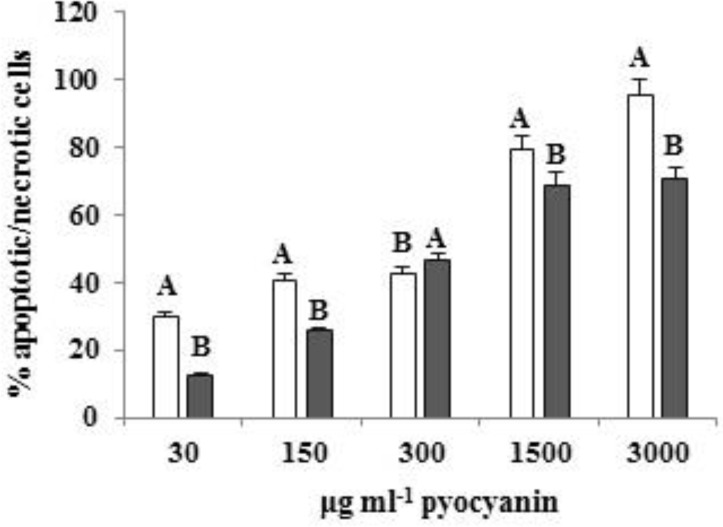
T-test analysis of the comparison between effects of C_11_ and E_8_ pyocyanin on the induction of apoptosis and/or necrosis in Panc-1 cells. The percentage of apoptotic/necrotic cells exhibited greater results in all doses of C_11_ except 300 μl.ml-1. Several letters depict between-group significant differences (*P* < 0.01)

Flow cytometric results also showed that both studied pyocyanins can induce concentration-dependent apoptosis/necrosis in Panc-1 cells; the higher the concentration, the greater the induction. Accordingly, 3 mg.ml^-1^ of C_11_ pyocyanin (the highest concentration) led to apoptosis/necrosis in 95.25% of Panc-1 cells and the same concentration of E_8_ pyocyanin-induced apoptosis/necrosis in 70.49% of cells, whereas the lowest concentration of C_11 _and E_8_ pyocyanin (30 μg.ml^-1^) induced apoptosis/necrosis in 29.96 and 12.49% of cells, respectively (Figures 5, 6). Table 1 shows the proportion of apoptotic and/or necrotic cells.

## Discussion

*P. aeruginosa* strains produce several redox-active compounds as secondary metabolites. The best-studied one, pyocyanin, is a blue-green pigment soluble in chloroform and plays a key role in virulence of *P. aeruginosa *(16, 17). In pyocyanin biosynthesis pathway, the modification of phenazine-1-carboxylic acid to pyocyanin consists of N-methylation of PCA via PhzM followed by the reduction of carboxylic acid group catalyzed by PhzS. PhzS can also oxidize PCA to give the final yellowish product named 1-hydroxyphenazine (18).

To confirm pyocyanin production in studied isolates of *P. aeruginosa*, including C_11_ and E_8_ (greenish pigment producers) as well as C_1_ (yellowish pigment producer), PCR amplification of *phzM* was performed. Results revealed that greenish pigment producers represented the DNA fragment containing *phzM*, while no desired DNA fragment was observed in C_1_. Researchers have reported that both *phzM* and *phzS* genes are essential for the conversion of PCA to pyocyanin. Using a mutant derivative of *P. aeruginosa *PAO1 with mutation in *phzM*, they demonstrated that cultures of the mutant were yellow due to the production of 1-hydroxy-phenazine, whereas cultures of the wild-type PAO1 were blue due to pyocyanin production, concluding that PhzS catalyzes the production of 1-hydroxy-phenazine, and PhzS together with PhzM catalyzes pyocyanin biosynthesis (18).

To extract pyocyanin from selected clinical and soil isolates, the chloroform-acid method was used and extracted pigments were then characterized by analyses of their UV-Vis, FT-IR, and HPLC spectra against a standard pyocyanin. UV-Vis spectra of pyocyanin showed maxima absorption at 308 and 694 nm when dissolved in chloroform, whereas values were 387 and 518 nm when dissolved in 0.1 N HCl, which are consistent with results of previous studies (19, 20).

In the present study, 90 and 75 mg of pyocyanin were extracted from C_11_ and E_8_ *P. aeruginosa* isolates, respectively. Treatment of Panc-1 cells by C_11_ and E_8_ pyocyanin led to a highly concentration-dependent cell cytotoxicity and apoptosis/necrosis after 24 hr. Not only did the C_11_ isolate produce higher amounts of pyocyanin, but the cytotoxic effect of C_11_ pyocyanin on Panc-1 cells was also greater than E_8_ pyocyanin in all treatments with significant IC_50_ differences, indicating that clinical strains of *P. aeruginosa* might produce more active pyocyanin molecules. Results of the present study are consistent with previous ones despite some differences. Zhao *et al.* studied the proliferation of HepG2 cells in the presence of 10 μg.ml^-1^ pyocyanin. In their study, a nine-day treatment by pyocyanin led to a 67% decline in HepG2 cell numbers compared to the control. Results of their study demonstrated that cell death induced by pyocyanin arises from oxidative stress due to the ROS augmentation, damage to DNA, activation of caspase-3, and the acceleration of senescence and apoptosis (9). Moreover, another study showed that 450, 225, and 112.5 μg.ml^-1^ of pyocyanin resulted in %IR of 84.02±0.34, 79.09±0.59, and 75.67% in HepG2 after a 72 hr treatment, respectively (21). Hassani *et al.* also studied the cytotoxic effect of pyocyanin from mutant and wild-type strains of *P. aeruginosa* on RD cells. They have reported that after a 72 hr treatment by pyocyanin, the mutant studied strain resulted in higher levels of %IR than the wild type; respectively, %IR of 88 and 64% for the highest pyocyanin concentration (500 μg.ml^-1^) and 65 and 28% for the lowest (7.8125 μg.ml^-1^) as well as IC_50_ of 225 and 57.3 μg.ml^-1^ after a 48-hour treatment (10).

Redox activity plays the key role in pyocyanin cytotoxicity; pyocyanin is a zwitterion molecule that can be in oxidized (blue) and reduced (colorless) forms and easily crosses the cellular membrane. As pyocyanin enters a cell, it interacts directly with cellular NADH and NADPH resulting in ROS augmentation. The generated ROS can lead to oxidative stress in host cells and cell damage (22). In addition, most human cells exert several key mechanisms to bound destructive effects of ROS; one of which is glutathione. When cells are exposed to such oxidant species, reduced glutathione (GSH) is oxidized to a dimer [oxidized glutathione (GSSG)], which is accomplished by glutathione peroxidase. GSSG, however, is reduced back to GSH through the function of glutathione reductase and this GSH cycle appears to serve as the major procedure in limiting ROS cytotoxicity. Consumption and loss of cellular GSH can result in cellular exposure to oxidant agents and occurrence of oxidant-mediated cell damage (23). Pyocyanin has the capacity to accept one electron from GSH to generate pyocyanin radical; hence, it utilizes oxidant defense mechanism of target cells to generate ROS and gives a rise in oxidative invasion (24). As a result, the increase of ROS and the inhibition of anti-oxidant mechanisms by pyocyanin disturb the anti-oxidant ability of tissue and result in cell injury (25).

## Conclusion

In the current study, pyocyanin showed a high cytotoxic effect against Panc-1 cells and induced apoptosis/necrosis in these cells. Using animal models, the studies of anti-cancer effects of pyocyanin can be expanded in order to introduce it as a potential anti-cancer biodrug.

## References

[1] Lau GW, Hassett DJ, Ran H, Kong F (2004). The role of pyocyanin in Pseudomonas aeruginosa infection. Trends Mol Med.

[2] Muller M, Li Z, Maitz PK (2009). Pseudomonas pyocyanin inhibits wound repair by inducing premature cellular senescence: role for p38 mitogen-activated protein kinase. Burns.

[3] Budzikiewicz H (1993). Secondary metabolites from fluorescent pseudomonads. FEMS Microbiol Rev.

[4] Hassett D, Charniga L, Bean K, Ohman D, Cohen M (1992). Response of Pseudomonas aeruginosa to pyocyanin: mechanisms of resistance, antioxidant defenses, and demonstration of a manganese-cofactored superoxide dismutase. Infect Immun.

[5] Denning GM, Railsback MA, Rasmussen GT, Cox CD, Britigan BE (1998). Pseudomonas pyocyanine alters calcium signaling in human airway epithelial cells. Am J Physiol: Lung Cell Mol Physiol.

[6] Usher LR, Lawson RA, Geary I, Taylor CJ, Bingle CD, Taylor GW (2002). Induction of neutrophil apoptosis by the Pseudomonas aeruginosa exotoxin pyocyanin: a potential mechanism of persistent infection. J Immunol.

[7] Laursen JB, Nielsen J (2004). Phenazine natural products: biosynthesis, synthetic analogues, and biological activity. Chem Rev.

[8] Mavrodi DV, Blankenfeldt W, Thomashow LS (2006). Phenazine compounds in fluorescent Pseudomonas spp biosynthesis and regulation. Annu Rev Phytopathol.

[9] Zhao J, Wu Y, Alfred A, Wei P, Yang S (2014). Anticancer effects of pyocyanin on HepG2 human hepatoma cells. Lett Appl Microbiol.

[10] Hassani HH, Hasan HM, Al-Saadi A, Ali AM, Muhammad MH (2012). A comparative study on cytotoxicity and apoptotic activity of pyocyanin produced by wild type and mutant strains of Pseudomonas aeruginosa. Eur J Exp Biol.

[11] Lozano R, Naghavi M, Foreman K, Lim S, Shibuya K, Aboyans V (2013). Global and regional mortality from 235 causes of death for 20 age groups in 1990 and 2010: a systematic analysis for the Global Burden of Disease Study 2010. The Lancet.

[12] Al Haddad AH, Adrian TE (2014). Challenges and future directions in therapeutics for pancreatic ductal adenocarcinoma. Expert Opin Invest Drugs.

[13] Nakaike S, Yamagishi T, Nanaumi K, Otomo S, Tsukagoshi S (1992). Cell-killing activity and kinetic analysis of a novel antitumor compound NC-190, a benzo[a]phenazine derivative. Jpn J Cancer Res.

[14] Moayedi A, Nowroozi J, Akhavan Sepahy A (2017). Effect of fetal and adult bovine serum on pyocyanin production in Pseudomonas aeruginosa isolated from clinical and soil samples. Iran J Basic Med Sci.

[15] Gohain N, Thomashow LS, Mavrodi DV, Blankenfeldt W (2006). The purification, crystallization and preliminary structural characterization of PhzM, a phenazine-modifying methyltransferase from Pseudomonas aeruginosa. Acta Crystallogr Sect F Struct Biol Cryst Commun.

[16] Jayaseelan S, Ramaswamy D, Dharmaraj S (2014). Pyocyanin: production, applications, challenges and new insights. World J Microbiol Biotechnol.

[17] Ra’oof W, Latif I (2010). In vitro study of the swarming phenomena and antimicrobial activity of pyocyanin produced by Pseudomonas aeruginosa isolated from different human infections. Eur J Sci Res.

[18] Mavrodi DV, Bonsall RF, Delaney SM, Soule MJ, Phillips G, Thomashow LS (2001). Functional analysis of genes for biosynthesis of pyocyanin and phenazine-1-carboxamide from Pseudomonas aeruginosa PAO1. J Bacteriol.

[19] Ohfuji K, Sato N, Hamada-Sato N, Kobayashi T, Imada C, Okuma H (2004). Construction of a glucose sensor based on a screen-printed electrode and a novel mediator pyocyanin from Pseudomonas aeruginosa. Biosens Bioelectron.

[20] El-Fouly M, Sharaf A, Shahin A, El-Bialy HA, Omara A (2015). Biosynthesis of pyocyanin pigment by Pseudomonas aeruginosa. J Radiat Res Appl Sci.

[21] Mohammed HA, Yossef HS, Mohammad FI (2014). The cytotoxicity effect of pyocyanin on human hepatocellular carcinoma cell line (HepG2). Iraqi J Sci.

[22] O’Malley YQ, Reszka KJ, Spitz DR, Denning GM, Britigan BE (2004). Pseudomonas aeruginosa pyocyanin directly oxidizes glutathione and decreases its levels in airway epithelial cells. Am J Physiol: Lung Cell Mol Physiol.

[23] Dickinson DA, Forman HJ (2002). Cellular glutathione and thiols metabolism. Biochem Pharmacol.

[24] Jacob C, Jamier V, Ba LA (2011). Redox active secondary metabolites. Curr Opin Chem Biol.

[25] Wilson R, Sykes D, Watson D, Rutman A, Taylor G, Cole P (1988). Measurement of Pseudomonas aeruginosa phenazine pigments in sputum and assessment of their contribution to sputum sol toxicity for respiratory epithelium. Infect Immun.

